# Evaluating the effects of seed oils on lipid profile, inflammatory and oxidative markers, and glycemic control of diabetic and dyslipidemic patients: a systematic review of clinical studies

**DOI:** 10.3389/fnut.2025.1502815

**Published:** 2025-02-07

**Authors:** Lucas Fornari Laurindo, Lívia Fornari Laurindo, Victória Dogani Rodrigues, Jéssica da Silva Camarinha Oliveira, Beatriz Leme Boaro, Adriano Cressoni Araújo, Elen Landgraf Guiguer, Claudia Rucco Penteado Detregiachi, Virgínia Maria Cavallari Strozze Catharin, Eduardo Federighi Baisi Chagas, Vitor Cavallari Strozze Catharin, Rosa Direito, Sandra Maria Barbalho

**Affiliations:** ^1^Department of Biochemistry and Pharmacology, School of Medicine, Faculdade de Medicina de Marília (FAMEMA), Marília, São Paulo, Brazil; ^2^Department of Administration, Associate Degree in Hospital Management, Universidade de Marília (UNIMAR), Marília, São Paulo, Brazil; ^3^Department of Biochemistry and Pharmacology, School of Medicine, Universidade de Marília (UNIMAR), Marília, São Paulo, Brazil; ^4^Department of Biochemistry and Pharmacology, School of Medicine, Faculdade de Medicina de São José do Rio Preto (FAMERP), São José do Rio Preto, São Paulo, Brazil; ^5^Postgraduate Program in Structural and Functional Interactions in Rehabilitation, School of Medicine, Universidade de Marília (UNIMAR), Marília, São Paulo, Brazil; ^6^Department of Biochemistry and Nutrition, School of Food and Technology of Marília (FATEC), Marília, São Paulo, Brazil; ^7^Laboratory of Systems Integration Pharmacology, Clinical and Regulatory Science, Research Institute for Medicines, Universidade de Lisboa (iMed.ULisboa), Lisbon, Portugal; ^8^UNIMAR Charity Hospital, Universidade de Marília (UNIMAR), Marília, São Paulo, Brazil

**Keywords:** seed oils, dyslipidemia, type 2 diabetes, lipid profiles, inflammatory markers, glycemic control, clinical trials, cardiometabolic health

## Abstract

Diabetes mellitus and dyslipidemia are significant health concerns that elevate the risk of cardiovascular disease and other metabolic disorders, necessitating effective management strategies. Recent research has highlighted the potential role of dietary fats, particularly seed oils, in influencing health outcomes in these conditions. This systematic review evaluates the impact of seed oils on lipid profiles, inflammatory and oxidative markers, and glycemic control in patients with diabetes and dyslipidemia. A comprehensive search across databases, including PubMed, Scopus, Web of Science, Cochrane Library, and Google Scholar, identified studies focusing on the effects of seed oils. The studies include randomized controlled, parallel-design, double-blind, placebo-controlled, and open-label studies published in English. The quality of the studies was assessed through a detailed review process, and data were extracted to evaluate the effects of seed oils on key metabolic markers. The review included 11 studies demonstrating that seed oils derived from canola, flaxseed, and sesame seeds can positively influence lipid profiles and glycemic control while potentially modulating oxidative stress markers. The findings suggest that seed oils may benefit in managing diabetes and dyslipidemia, although the results are sometimes inconsistent. This review provides valuable insights for dietary recommendations and therapeutic strategies, highlighting the need for further research to clarify the role of seed oils in metabolic health.

## 1 Introduction

Diabetes mellitus and dyslipidemia are significant health concerns that contribute to an increased risk of cardiovascular disease and other metabolic disorders (1, 2). Both conditions require effective management strategies to mitigate their adverse effects on health (3, 4). In recent years, dietary interventions have emerged as a pivotal component of treatment plans for these conditions, focusing on the types of fats consumed (5–7). Seed oils have attracted considerable attention among various dietary fats due to their distinct fatty acid profiles and potential impact on health outcomes (8–10).

Seed oils, such as those derived from sunflower (11), safflower (12), and canola seeds (13), are commonly used in cooking and food preparation. They are often touted for their favorable fatty acid composition (14), including high levels of Polyunsaturated Fatty Acids (PUFAs) (15), which are believed to influence lipid profiles and other metabolic markers positively (16). However, the reports of the effects of these oils on lipid levels, inflammation, oxidative stress, and glycemic control in diabetic and dyslipidemic patients are complex and sometimes conflicting.

The detrimental effects of chronic inflammation and oxidative stress on health are well-documented (17, 18). Chronic inflammation has been linked to the progression of insulin resistance (19) and diabetes (20), contributing to the development of cardiovascular diseases and other serious complications (21, 22). Oxidative stress, resulting from an imbalance between reactive oxygen species and the body's ability to neutralize them, exacerbates inflammatory responses and damages cellular structures (23, 24), further impairing metabolic health and increasing disease risk (25). These processes play a crucial role in the pathophysiology of diabetes and dyslipidemia (26–29), underscoring the importance of dietary factors that can modulate these harmful effects (30, 31).

Despite the growing body of research on seed oils, there has yet to be a comprehensive systematic review that consolidates the evidence explicitly focusing on their impact on diabetic and dyslipidemic patients. Existing reviews often address broader dietary fat topics or focus on single health outcomes, lacking a focused analysis of seed oils across multiple metabolic markers. Additionally, there is a limited synthesis of how seed oils influence inflammatory and oxidative stress pathways in these conditions. Tian et al. (32) discussed the health-promoting effects of vegetable oils, highlighting their chemical compositions and pharmacological potential. However, their comprehensive analysis relied majorly on chemical compositions and nutritional values and did not evaluate the health benefits systematically and holistically. In other words, they did not focus on the analysis of the included studies but on the oil's characteristics, with prospecting results mainly based on their bioactive components. In addition, they did not solely focus on diabetes and dyslipidemia patients but on cancer and individuals suffering from cardiovascular disease. On the other hand, Schwingshackl et al. (33) published a systematic review focusing on the effects of oils and solid fats on blood lipids. Although their analysis was comprehensive, they did not focus solely on dyslipidemic patients and lacked comparisons on the effects of vegetable oils in patients suffering from diabetes and dyslipidemia together. In addition, they did not focus only on seed oils but also on solid fats. Finally, none of the studies mentioned above analyzed the effects of seed oils on inflammatory and oxidative markers during their interventions with diabetic and dyslipidemic patients. Since inflammation and oxidative stress are paramount components of diabetes and dyslipidemia pathophysiologies and related pathologies, our study is of utmost importance since we analyzed patients suffering from these two conditions and their respective markers.

To address the existing knowledge gap, this systematic review comprehensively evaluates clinical studies that assess the impact of seed oils on key health markers in patients with diabetes and dyslipidemia. By synthesizing data from diverse studies, this review aims to elucidate how seed oils influence various health parameters, including lipid profiles, inflammatory and oxidative markers, and glycemic control. The insights derived from this analysis are expected to inform dietary recommendations and therapeutic strategies, thereby contributing to improved management and outcomes for individuals affected by these common conditions. It also highlights the limitations of the included studies, such as variability in study designs, sample sizes, and methods of assessing health outcomes. By identifying these limitations, the review underscores the need for more robust, longitudinal, and methodologically sound research to clarify the relationship between seed oils and health markers. Addressing these research gaps will be crucial for developing more accurate and evidence-based dietary guidelines and therapeutic approaches.

## 2 Literature search methodology

In this section, we outline the systematic approach taken to identify and analyze relevant studies concerning the impact of seed oils on dyslipidemia and type 2 diabetes. The comprehensive search strategy was designed to ensure a thorough and unbiased review of the existing literature.

### 2.1 Databases searched

To identify relevant studies, a comprehensive search was conducted across the following electronic databases: PubMed, Scopus, Web of Science, Cochrane Library, and Google Scholar. These databases were chosen to ensure broad coverage and include peer-reviewed journals and gray literature.

### 2.2 Search strategy

The search strategy involved using specific keywords and their combinations to capture studies on seed oils' impact on dyslipidemia and type 2 diabetes. The primary keywords included “seed oils,” “dyslipidemia,” “hyperlipidemia,” “type 2 diabetes,” “lipid profiles,” “inflammatory markers,” “glycemic control,” and “clinical trials.” These keywords were combined using Boolean operators (AND, OR) to refine the search results. For instance, combinations like “seed oils AND dyslipidemia,” “seed oils AND type 2 diabetes,” and “seed oils AND lipid profiles” were used.

### 2.3 PRISMA guidelines

The Preferred Reporting Items for Systematic Reviews and Meta-Analyses (PRISMA) (34) guidelines were followed to ensure a systematic and transparent approach to the literature search and selection process. The PRISMA flow diagram was used to document the number of studies identified, screened, assessed for eligibility, and included in the review, along with reasons for exclusion at each stage.

### 2.4 Inclusion and exclusion criteria

Studies were included if they were randomized controlled trials, cohort studies, or case-control studies examining the effects of seed oils on lipid profiles, inflammatory markers, or glycemic control in patients with dyslipidemia or type 2 diabetes. Studies had to be published in English and involve human participants. Exclusion criteria included review articles, animal studies, studies unrelated to the specified seed oils, and studies without relevant outcome measures.

### 2.5 Quality assessment

The quality of the included studies was assessed through a comprehensive review process following the Cochrane Handbook for Systematic Reviews of Interventions (35). A detailed evaluation was conducted for randomized and non-randomized controlled trials to identify potential biases such as selection, performance, detection, attrition, and reporting. This involved carefully examining how participants were selected, how interventions were administered, and how outcomes were measured and reported. This assessment aimed to ensure that the studies appropriately selected participants, matched them to relevant variables and accurately reported outcomes. Two researchers (Lucas. F.L. and S.M.B.) independently reviewed each study to ensure a rigorous evaluation process. In cases where disagreements arose between the reviewers, these were resolved through discussion or consulting a third reviewer (R.D.) to achieve consensus. The selection bias involves evaluating studies with unclear or high selection bias for individual inclusions in each interventional or non-interventional group. Randomization and allocation methods are determined and discussed based on each study design to ensure the utmost accuracy of the included findings. Secondly, the performance and detection bias report involves studies lacking blinding, which might impact the studies' results based on their specific begins. Thirdly, attrition and reporting bias involve attrition rates or selective reporting of results in the included studies. Studies missing data or skewing results must be excluded. For a study to be included besides bias recognition, it must not have had critical bias reporting. This approach aimed to enhance the reliability and validity of the quality assessment studies included in the review.

### 2.6 Data extraction and synthesis

Two experienced researchers (Lucas. F.L. and S.M.B.) extracted data from the included studies. Following data collection, two additional researchers (Lívia. F.L. and R.D) verified the data's significance, reliability, and correctness. Relevant studies were screened based on titles and abstracts, followed by a full-text review. Data extracted included study design, sample size, participant characteristics, type and dosage of seed oil used, duration of intervention, and outcomes related to lipid profiles, inflammatory markers, and glycemic control. The extracted data were synthesized to evaluate the overall impact of seed oils on dyslipidemia and type 2 diabetes, highlighting the most significant findings and trends. The results were presented in a narrative format, supported by tables and figures where appropriate.

By employing this methodology, the review aims to provide a comprehensive understanding of the therapeutic potential of various seed oils in managing dyslipidemia and type 2 diabetes. This systematic approach ensures the reliability and validity of the findings, contributing valuable insights to the field of nutritional therapy.

## 3 Results of literature search methodology and overview of the included studies

This section outlines the results of our literature search methodology and provides an overview of the studies included in the review. It presents a comprehensive evaluation of the effects of seed oils on dyslipidemia and type 2 diabetes.

### 3.1 Literature search results

**Figure 1:**
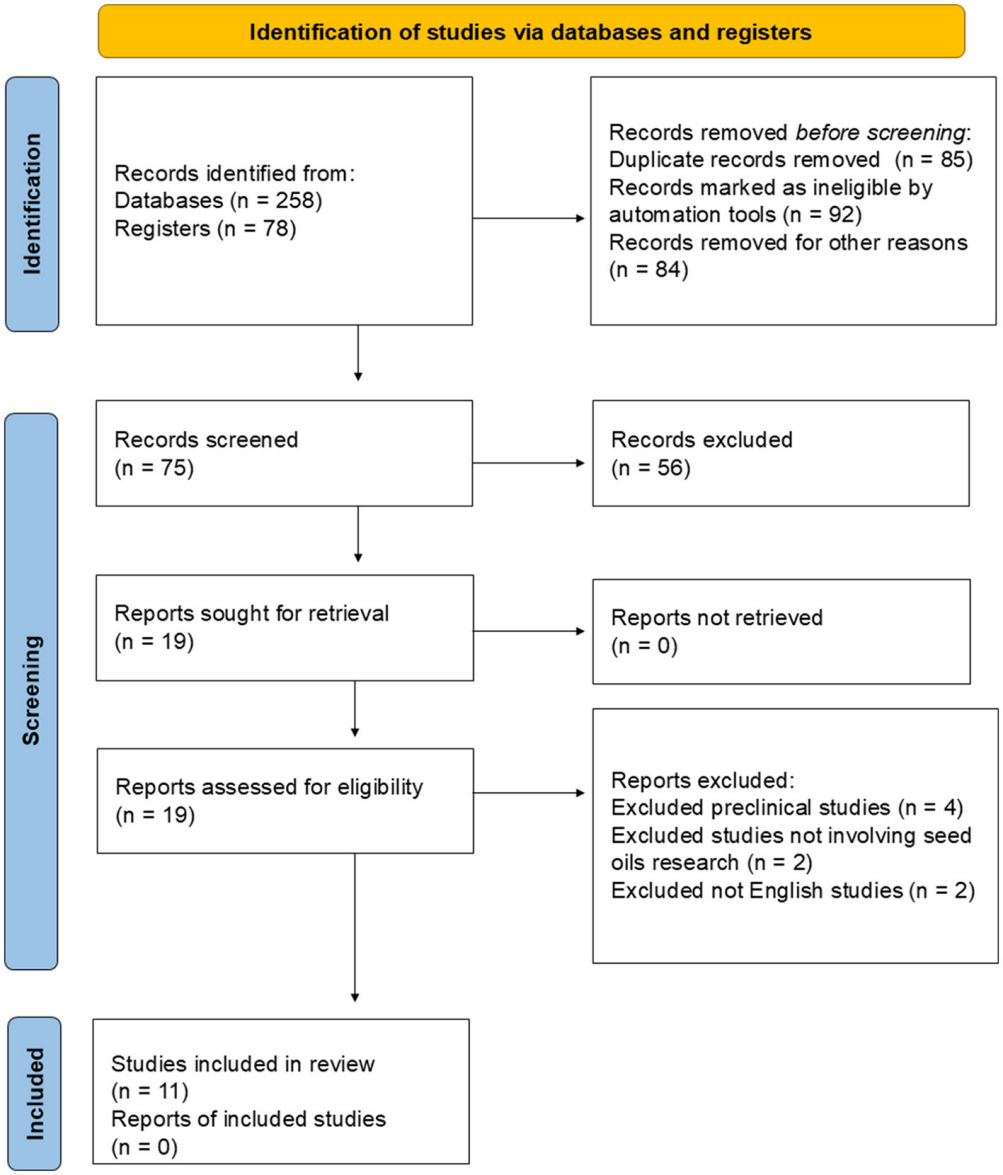
Process flow for record selection and inclusion in the review following PRISMA.

The initial search process produced a comprehensive dataset of 258 records from various databases and an additional 78 records from registries. Following this initial collection, duplicate records were identified and removed, totaling 85 duplicates. In addition to eliminating these duplicates, ineligible records flagged by automation tools (Rayyan online application) were also excluded, accounting for 92 records. Moreover, 84 records were excluded for other reasons unrelated to eligibility criteria, 70 still duplicate publications, and 14 studies of non-peer-reviewed sources). After applying these exclusions, 75 records remained for the screening phase. During the screening process, 56 records were excluded based on their content, which did not meet the criteria for inclusion in the review. Subsequently, 19 reports were selected for retrieval and thorough assessment. Each of these 19 reports was successfully retrieved. However, upon evaluation for eligibility, 8 of these reports were excluded: 4 were preclinical studies not relevant to the research question, two did not focus on seed oils as required, and two were in languages other than English, which were not feasible for inclusion. As a result of this assessment, 11 studies were deemed suitable and were included in the final review. No additional reports from the included studies were missing from the final assessment process. The PRISMA (Figure 1) flow diagram illustrates the study selection process, including the reasons for exclusion at each stage. All studies underwent a rigorous quality assessment using the COCHRANE handbook for intervention evaluations. This assessment evaluated selection, performance, detection, attrition, and reporting bias. It is worth noting that, following our quality assessment, no studies were excluded for bias reporting, meaning that all included studies achieved minimum quality standards set out in our inclusion criteria.

### 3.2 Overview of the included studies

Table 1 provides a comprehensive overview of clinical studies evaluating the impact of various oil supplements on glycaemic control, lipid profiles, and inflammatory and oxidative markers. The table summarizes key aspects of each study, including population characteristics, intervention details, comparisons, primary outcome measures, and notable results. Table 2 provides the COCHRANE assessment for the included interventions. Selection, performance, detection, attrition, and reporting biases were reported using “critical,” “serious,” “moderate,” or “low” bias risk stamps.

**Table 1:** Summary of clinical studies on oil supplementation and its effects on glycemic control, lipid profiles, and inflammatory and oxidative markers.

**Study**	**Population**	**Intervention**	**Comparison**	**Outcome measures**	**Glycemic control**	**Lipid profiles**	**Inflammatory markers**	**Oxidative stress markers**
Nikooyeh et al. (36)	92 adults with T2DM, aged 20–65 years, not receiving insulin, recruited from Iran Diabetes Society and the general population, with BMI 28.4 ± 0.79 for Group 1, 29.7 ± 0.72 for Group 2, and 29.2 ± 0.68 for Group 3	ORZ-fortified canola oil (Group 1, *n =* 30) for 12 weeks; unfortified canola oil (Group 2, *n =* 32) for 12 weeks; SO (Group 3, *n =* 30) for 12 weeks; Randomized, double-blind clinical trial	Unfortified canola oil (*n =* 32) and SO (*n =* 30) for 12 weeks	**Primary:** WC, BP, FBG, HbA1c, TG	Significant reductions in FBG (134.0 ± 6.2 mg/dL → 126.4 ± 5.8 mg/dL) and HbA1c (6.1% ± 0.12% → 5.4% ± 0.12%) in ORZ-fortified canola oil group only.	There was a significant decrease in TG (134.7 ± 9.8 mg/dL → 116.8 ± 7.8 mg/dL) only in the ORZ-fortified canola oil group. A reduction in TG was observed in all groups but not substantial for unfortified canola oil (147.1 ± 11.8 mg/dL → 129.8 ± 10.5 mg/dL) or SO (131.1 ± 10.5 mg/dL → 115.7 ± 7.7 mg/dL).	Not reported.	Not reported.
Haldar et al. (39)	143 borderline hypercholesterolemic Chinese volunteers, aged 50–70 years, with BMI ≤ 27.5 kg/m^2^	30 g of refined rice bran, flaxseed, and sesame oil blends per day for 8 weeks (*n =* 88); Parallel-design, randomized controlled trial	Refined olive oil (*n =* 47)	**Primary:** Serum TC, LDL-C, TG, apoB, TC to HDL-C ratio, apoB to apoA1 ratio; **Secondary:** Systolic and diastolic BP, serum glucose, body weight	Significant reductions in FBG levels (−1.51%).	Significant reductions in serum TC (−3.47%), LDL-c (−4.16%), TG (−10.3%), apoB (−3.93%), TC to HDL-C ratio (−3.44%), apoB to apoA1 ratio (−3.99%), systolic and diastolic BP (−3.32% and −3.16%); Small but significant increase in body weight; No significant effects on HDL-C or apoA1 concentration.	Not reported.	Not reported.
Aslam et al. (38)	46 adults (18–60 years) with T2DM from Pakistan	30 ml of white sesame seed oil daily for 90 days; Randomized controlled trial	30 ml of soybean oil daily for 90 days (control group)	**Primary:** Blood glucose, insulin, HbA1c, TBARS, SOD, CAT, GPx	Blood glucose decreased in the DSO group (189.09 ± 4.42 mg/dL → 137.8 3 ± 3.16 mg/dL) and increased in the DCON (185.04 ± 6.84 mg/dL → 218.14 ± 5.92 mg/dL) group. Insulin was higher in the DSO group (12.26 ± 1.24 μU/mL → 23.13 ± 1.15 μU/ml) than in the DCON group (11.97 ± 0.81 μU/mL → 7.93 ± 0.38 μU/mL). HbA1c was lower in the DSO (6.96% ± 0.26%) group at 90 days compared to the DCON group (8.02% ± 0.37%).	Not reported.	Not reported.	TBARS decreased significantly in the DSO group (1.91 ± 0.00 nmol/mL → 1.08 ± 0.05 nmol/mL) and increased in the DCON group (1.82 ± 0.01 nmol/mL → 2.26 ± 0.07 nmol/mL). SOD (2.79 ± 0.02 U/mL → 4.91 ± 0.10 U/mL), CAT (5.18 ±0.04 U/mL → 6.41 ± 0.06 U/mL), and GPx (141.55 ± 0.12 U/mL → 147.14 ± 0.17 U/mL) increased in the DSO group, indicating improved antioxidant activity, while these markers decreased in the DCON group (SOD: 2.81 ± 0.02 U/mL → 2.34 ± 0.30 U/mL, CAT: 5.14 ± 0.04 U/mL → 3.42 ± 0.05 U/mL, GPx: 139.06 ± 1.24 U/mL → 101.97 ± 1.80 U/mL).
Khajebishak et al. (42)	60 obese type 2 diabetic patients (both genders), aged 30–50 years, BMI >30 and < 40 kg/m^2^ from Iran	PSO capsules (*n =* 26) (1 g/day) for 8 weeks; Randomized controlled clinical trial	Placebo capsules (*n =* 26) (paraffin) for 8 weeks	**Primary:** FBG, HbA1c, Insulin, HOMA-IR, QUICKI, GLUT-4 Gene Expression	GLUT-4 gene expression increased significantly in the PSO group compared to the placebo group. FBG decreased substantially in the PSO (161.46 ± 34.44 mg/dL → 143.50 ± 24.2 mg/dL) group, with no significant change in the placebo group (156.54 ± 31.90 mg/dL → 154.65 ± 31.48 mg/dL); HbA1c showed a slight decrease in the PSO group (7.53% ± 0.92% → 7.25% ± 0.80%) with no significant change in the placebo group (7.65% ± 1.07% → 7.52% ± 1.04%). QUICKI improved significantly in the PSO group (0.30 ± 0.02 → 0.31 ± 0.03); there was no change in the placebo group.	Not reported.	Not reported.	Not reported.
Akrami et al. (45)	60 adults (30–60 years) with MetSyn in Iran, with 81.17 kg ± 11.23 kg for FO and 84.50 ± 14.89 kg for SO	25 mL/day FO for 7 weeks (*n =* 30); Randomized controlled trial	25 mL/day SO for 7 weeks (*n =* 30)	**Primary:** BP, serum lipids, FBG, MDA	There is no significant between-group difference in FBG. However, significant within-group reductions in FBG were observed in FO (95.31 mg/dL ± 20.40 mg/dL → 90.46 mg/dL ± 21.90 mg/dL) and SO (93.96 mg/dL ± 23.18 mg/dL → 93.85 mg/dL ± 42.54 mg/dL) groups.	There were significant reductions in TC (FO, 215.15 mg/dL ± 42.74 mg/dL → 196.92 mg/dL ± 36.91 mg/dL; SO, 215.54 mg/dL ± 30.18 mg/dL → 190.35 mg/dL ± 33.34 mg/dL), LDL-C (FO, 128.23 mg/dL ± 29.80 mg/dL → 121.04 mg/dL ± 26.88 mg/dL; SO, 132.08 mg/dL ± 25.01 mg/dL → 117.73 mg/dL ± 27.63 mg/dL), and TG (FO, 209.27 mg/dL ± 106.34 mg/dL → 156.81 mg/dL ± 65.69 mg/dL; SO, 196.00 mg/dL ± 76.18 mg/dL → 142.54 mg/dL ± 66.07 mg/dL) within both groups.	Not reported.	There was a marginally significant reduction in MDA in the FO group (7.01 nmol/dL ± 1.70 nmol/dL → 5.59 nmol/dL ± 0.59 nmol/dL) vs. a non-significant reduction in the SO group (6.64 nmol/dL ± 0.94 nmol/dL → 6.12 nmol/dL ± 1.03 nmol/dL).
Jenkins et al. (37)	141 adults with T2DM (HbA1c 6.5%−8.5%) treated with oral antihyperglycemic agents in Canada, with 31 ± 6 kg/m^2^ for control and 30 ± 5 kg/m^2^ for test diets	Low-GL diet with 31 g canola oil/day (*n =* 70) provided as a supplement (4.5 slices of canola oil–enriched bread daily) for 3 months; Randomized, parallel design clinical trial	A whole-grain diet with 7.5 slices/day (*n =* 71) of whole-wheat bread	**Primary:** HbA1c; **Secondary:** Framingham CVD risk score, RHI	HbA1c significantly decreased in the test diet (−5.15 mmol/mol [−5.92 mmol/mol, −4.38 mmol/mol]). There are more significant benefits in those with higher systolic BP.	Test diet led to significant reductions in TC (−0.30 mmol/L [−0.38 mmol/L, −0.22 mmol/L]), LDL-C (−0.20 mmol/L [−0.27 mmol/L, −0.13 mmol/L]), TG (−0.15 mmol/L [−0.24 mmol/L, −0.07 mmol/L]), and HDL-C (−0.03 mmol/L [−0.05 mmol/L, −0.01 mmol/L]) compared to control diet (TC, 0.04 mmol/L [−0.03 mmol/L, 0.12 mmol/L]; LDL-C, 0.04 mmol/L [−0.02 mmol/L, 0.11 mmol/L]; TG, −0.01 mmol/L [−0.09 mmol/L, 0.07 mmol/L]; HDL-C, 0.00 mmol/L [−0.02 mmol/L, 0.02 mmol/L]).	Not reported.	Not reported.
Wei et al. (43)	Men and women aged 18–75 with elevated blood lipids (*n =* 36) from China	Perilla oil capsules (4 capsules, twice daily for 8 weeks) + exercise (30–60 min/day, at least 4 days/week for 8 weeks) (*n =* 12); Prospective, randomized control trial involving men	EG (*n =* 12, 30–60 min per day, at least 4 days per week), MG (*n =* 12, four capsules, taken twice a day)	**Primary:** TC, TG, HDL-C, LDL-C, hs-CRP, PAI-1, TNF-α	Not reported.	Significant reduction in TC (6.2 ± 0.7 mmol/L → 5.3 ± 0.9 mmol/L), TG (3.4 ± 0.8 mmol/L → 2.8 ± 0.7 mmol/L), and LDL-C (4.0 ± 0.8 mmol/L → 3.1 ± 0.7 mmol/L) in EMG compared to EG and MG after 56 days. HDL-C increased significantly in MG (1.4 ± 0.4 mmol/L → 1.6 ± 0.6 mmol/L) and EMG (1.4 ± 0.4 mmol/L → 1.6 ± 0.6 mmol/L).	hs-CRP (3.43 mg/L ± 0.58 mg/L → 2.76 mg/L ± 0.54 mg/L), PAI-1 (37.79 ng/mL ± 5.98 ng/mL → 33.89 ng/mL ± 5.93 ng/mL), and TNF-α (1.23 ng/mL ± 0.19 ng/mL → 0.88 ng/mL ± 0.21 ng/mL) levels significantly reduced in EMG compared to EG (hs-CRP, 3.41 mg/L ± 0.63 mg/L → 2.74 mg/L ± 0.53 mg/L; PAI-1, 38.87 ng/mL ± 6.18 ng/mL → 33.56 ng/mL ± 5.88 ng/mL; TNF-α, 1.21 ng/mL ± 0.19 ng/mL → 0.97 ng/mL ± 0.18 ng/mL) and MG (hs-CRP, 3.38 mg/L ± 0.55 mg/L → 2.77 mg/L ± 0.61 mg/L; PAI-1, 39.24 ng/mL ± 6.23 ng/mL → 34.19 ng/mL ± 6.12 ng/mL; TNF-α, 1.23 ng/mL ± 0.24 ng/mL → 0.94 ng/mL ± 0.22 ng/mL).	Not reported.
Alipoor et al. (40)	Hyperlipidemic patients (*n =* 38) from Iran aged 50–70 years with BMI 18.5–30 kg/m^2^, total plasma cholesterol >200 mg/dL or total plasma TG >150 mg/dL, and on medical treatment for >3 months	White sesame seeds (40 g/day for 60 days) + 240 kcal removed from diet; 60 days treatment duration (*n =* 19); Randomized control trial	The control group receiving the same drug treatments without sesame seeds (*n =* 19)	**Primary:** Lipid profile (TC, LDL-C, TC/HDL-C ratio); Antioxidant markers (GPx, SOD, TBARS); Anthropometric measurements (Weight, BMI)	Not reported.	Significant decrease in TC (241.2 mg/dL ± 41.2 mg/dL → 221.5 mg/dL ± 45.2 mg/dL), LDL-C (159.7 mg/dL ± 37.8 mg/dL → 144.0 mg/dL ± 43.7 mg/dL), and TC/HDL-C ratio (5.2 ± 1.1 → 4.9 ± 1.2).	Not Reported.	Decreased TBARS (2.9 μmol/L ± 1.0 μmol/L → 1.9 μmol/L ± 1.0 μmol/L), increased GPx (21.4 U/Hb(g) ± 2.2 U/Hb(g) → 22.5 U/Hb(g) ± 2.0 U/Hb(g)) and SOD (1754.9 U/Hb(g) ± 269.9 U/Hb(g) → 1890.9 U/Hb(g) ± 308.4 U/Hb(g)).
Asghari et al. (44)	Males and females (*n =* 51), aged ≥20 years from Iran with BMI ≤ 35 kg/m^2^, serum TC >200 mg/dL, serum TG >150 mg/dl	PSO (800 mg/day, *n* = 25) vs. placebo (800 mg paraffin/day) for 4 weeks; Double-blind, randomized, placebo-controlled clinical trial	Placebo (*n =* 26)	**Primary:** Serum TG, HDL-C; TNF-α; TG/HDL-C ratio	Not reported.	Reduced serum TG (3.45 mmol/L ± 1.56 mmol/L → 2.75 mmol/L ± 1.40 mmol/L) and TG/HDL-C (7.49 ± 4.95 → 5.73 ± 4.55) ratio with PSO; HDL-C (1.25 mmol/L ± 0.39 mmol/L → 1.38 mmol/L ± 0.44 mmol/L) increased with PSO.	No significant changes in TNF-α levels (14.73 pg/mL ± 5.25 pg/mL → 13.28 pg/mL ± 3.79 pg/mL).	Not reported.
Sankar et al. (41)	60 adults with T2DM, mean aged 57–58 years from India	Combination of sesame oil (35 g/day) + glibenclamide (5 mg/day) for 60 days (*n =* 20); Open-label study	Glibenclamide (*n =* 22, 5 mg/day, single dose) for 60 days; Sesame oil (*n =* 18, 35 g/day) for 60 days	**Primary:** Glycemic Control (Blood glucose, HbA1c); Lipid Profiles (TC, LDL-C, TG, HDL-C); Antioxidant activity	Combination therapy significantly reduced glucose (−36%) and HbA1c (−43%).	Substantial decreases in TC, LDL-c, and TG in sesame oil (20%, 33.8%, and 14%, respectively) and combination therapy (22%, 38%, and 15%, respectively); HDL-C increased significantly in sesame oil (+15.7%) and combination therapy (+17%).	Not reported.	Significant improvement in antioxidant activities with sesame oil and combination therapy (*p* < 0.001).
Mirmiran et al. (46)	Hyperlipidemic subjects (*n =* 51) from Iran, aged over 20 years, without allergy or liver dysfunction, BMI ≤ 35 kg/m^2^, serum TC >5.2 mmol/l, and TG >1.65 mmol/l	PSO (400 mg, twice daily) for 4 weeks (*n =* 25); Double-blind placebo-controlled clinical trial	Placebo (*n =* 26)	**Primary:** Serum TG, HDL-C	There were no significant changes in insulin/glucose concentrations between PSO (0.08 ± 0.04 → 0.09 → 0.04) and control groups (0.08 ± 0.04 → 0.07 ± 0.03).	Reduction in TG (3.45 mmol/L ± 1.56 mmol/L → 2.75 mmol/L ± 1.40 mmol/L) and TG/HDL-C ratio (7.49 ± 4.95 → 5.73 ± 4.55) with PSO; Cholesterol/HDL-C ratio decreased with PSO (5.87 ± 1.67 → 5.45 ± 1.51). HDL-C increased in the PSO group (1.25 mmol/L ± 0.39 mmol/L → 1.38 mmol/L ± 0.44 mmol/L) compared to placebo (1.27 mmol/L ± 0.23 mmol/L → 1.25 mmol/L ± 0.26 mmol/L). There were no significant changes in serum cholesterol and LDL-C concentrations.	Not reported.	Not reported.

apoA1, Apolipoprotein A1; apoB, Apolipoprotein B; BMI, Body Mass Index; BP, Blood Pressure; CAT, Catalase; CVD, Cardiovascular Disease; DCON, Diabetic Control Group; DSO, Diabetic Sesame Oil Group; EG, Exercise Group; EMG, Exercise and Medicine Group; FBG, Fasting Blood Glucose; FO, Flaxseed Oil; GL, Glycemic Load; GLUT-4, Glucose Transporter Type 4; GPx, Glutathione Peroxidase; HbA1c, Glycated Hemoglobin; HDL-C, High-Density Lipoprotein Cholesterol; HOMA-IR, Homeostatic Model Assessment of Insulin Resistance; hs-CRP, High-Sensitivity C-Reactive Protein; LDL-C, Low-Density Lipoprotein Cholesterol; MDA, Malondialdehyde; MetSyn, Metabolic Syndrome; MG, Medicine Group; ORZ, γ-Oryzanol; PAI-1, Plasminogen Activator Inhibitor-1; PSO, Pomegranate Seed Oil; QUICKI, Quantitative Insulin Sensitivity Check Index; RHI, Reactive Hyperemia Index; SOD, Superoxide Dismutase; SO, Sunflower Oil; T2DM, Type 2 Diabetes Mellitus; TAG, Triglycerides; TBARS, Thiobarbituric Acid Reactive Substances; TC, Total Cholesterol; TG, Triglycerides; TNF-α, Tumor Necrosis Factor-α; WC, Waist Circumference.

**Table 2:** Reporting of bias assessment based on selection, performance, detection, attrition, and reporting bias following the COCHRANE handbook for interventions assessment.

**Study**	**D1**	**D2**	**D3**	**D4**	**D5**	**D6**	**D7**	**Overall**
Nikooyeh et al. (36)	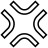	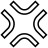	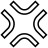	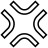	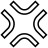	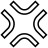	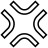	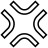
Haldar et al. (39)	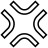	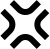	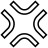	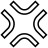	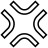	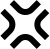	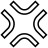	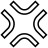
Aslam et al. (38)	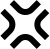	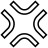	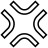	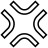	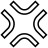	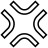	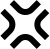	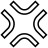
Khajebishak et al. (42)	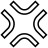	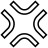	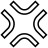	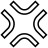	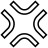	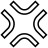	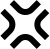	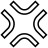
Akrami et al. (45)	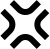	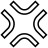	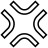	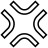	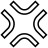	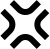	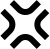	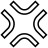
Jenkins et al. (37)	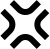	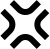	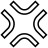	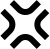	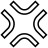	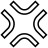	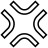	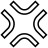
Wei et al. (43)	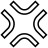	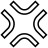	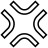	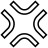	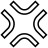	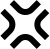	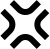	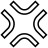
Alipoor et al. (40)	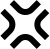	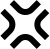	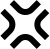	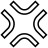	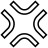	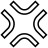	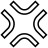	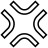
Asghari et al. (44)	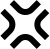	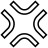	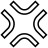	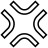	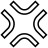	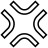	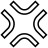	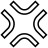
Sankar et al. (41)	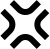	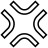	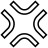	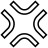	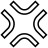	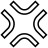	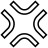	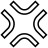
Mirmiran et al. (46)	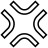	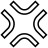	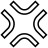	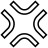	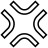	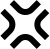	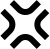	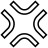

D1, bias due to confounding; D2, bias due to selection of participants; D3, bias in classification of interventions; D4, bias due to deviations from intended interventions; D5, bias due to missing data; D6, bias in measurement of outcomes; D7, bias in selection of the reported result. 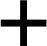, critical; 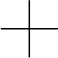, serious; 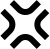, moderate; 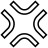, low.

The publication date range from the included studies is from 2010 to 2023. Regarding country examination, Iran possesses most of the included studies (*n* = 6), followed by China (*n* = 2), Pakistan (*n* = 1), Canada (*n* = 1), and India (*n* = 1). The most common seed oils utilized were canola oil, studied by Nikooyeh et al. (36) and Jenkins et al. (37), sesame oil, studied by Aslam et al. (38), Haldar et al. (39), Alipoor et al. (40), and Sankar et al. (41), perilla oil studied by Khajebishak et al. (42), Wei et al. (43), and Asghari et al. (44), Flaxseed Oil (FO) studied by Haldar et al. (39), rice bran oil studied by Haldar et al. (39), and Sunflower Oil (SO) studied by Nikooyeh et al. (36). The studies collectively explore how various oils and dietary modifications affect health outcomes such as glycemic control, lipid profiles, and oxidative stress markers. In this overview, we have grouped the results by outcomes following PRISMA guidelines recommendations.

The studies included in this research were conducted across various countries, each focusing on different health conditions and populations. Nikooyeh et al. (36) investigated 92 adults with type 2 diabetes from Iran. Haldar et al. (39) examined 143 borderline hypercholesterolemic Chinese volunteers. Aslam et al. (38) focused on 46 adults with type 2 diabetes from Pakistan. Khajebishak et al. (42) studied 60 obese type 2 diabetic patients in Iran. Akrami et al. (45) looked at 60 adults with Metabolic Syndrome (MetSyn), also from Iran. Jenkins et al. (37) conducted their trial with 141 adults with type 2 diabetes in Canada. Wei et al. (43) included 36 individuals with elevated blood lipids from China. Alipoor et al. (40) researched 38 hyperlipidemic patients from Iran. Asghari et al. (44) studied 51 individuals with specific lipid profiles from Iran. Sankar et al. (41) worked with 60 type 2 diabetes patients in India, and Mirmiran et al. (46) examined 51 hyperlipidemic subjects from Iran.

The participants' ages varied significantly in the studies. Nikooyeh et al. (36) included adults aged 20–65, while Haldar et al. (36) focused on individuals aged 50–70. Aslam et al. (38) studied adults between 18 and 60 years old. Khajebishak et al. (42) investigated patients aged 30–50 years. Akrami et al. (45) included adults aged 30–60 years. Jenkins et al. (37) worked with adults, although their age range was not detailed. Wei et al. (43) examined individuals from 18 to 75 years old. Alipoor et al. (40) studied participants aged 50–70 years. Asghari et al. (44) involved adults aged 20 years and older. Sankar et al. (41) had a mean participant age of 57–58 years, and Mirmiran et al. (46) included subjects over 20 years old.

#### 3.2.1 Glycemic and lipid profile controls

Nikooyeh et al. (36) found that γ-Oryzanol (ORZ)-fortified canola oil significantly improved fasting blood glucose and triglycerides. Haldar et al. (39) observed notable cholesterol and blood glucose reductions with a blend of rice bran, flaxseed, and sesame oils. Aslam et al. (38) reported that white sesame seed oil improved glycemic control, while Khajebishak et al. (42) found that Pomegranate Seed Oil (PSO) enhanced Glucose Transporter Type 4 (GLUT-4) gene expression and reduced fasting blood glucose. Akrami et al. (45) demonstrated reduced cholesterol and triglycerides with FO. Wei et al. (43) highlighted significant improvements in lipid profiles with perilla oil combined with exercise. Jenkins et al. (37) showed that a low-Glycemic Load (GL) diet with canola oil led to more significant reductions in Glycated Hemoglobin (HbA1c) and improvements in lipid profiles compared to a whole-grain diet. Alipoor et al. (40) found that white sesame seeds and dietary modifications significantly reduced total cholesterol and Low-Density Lipoprotein Cholesterol (LDL-C). Asghari et al. (44) observed reductions in triglycerides and the triglyceride/High-Density Lipoprotein Cholesterol (HDL-C) ratio with PSO. Sankar et al. (41) noted that combining sesame oil and glibenclamide substantially improved glycemic control and lipid profiles. Mirmiran et al. (46) reported that PSO reduced triglycerides and improved HDL-C levels.

#### 3.2.2 Inflammatory and oxidative markers

Aslam et al. (38) reported that sesame seed oil improves the body's antioxidant activity, while Wei et al. (43) highlighted significant improvements in inflammatory markers with perilla oil combined with exercise. Asghari et al. (44) observed insignificant reductions in inflammatory markers with PSO. Sankar et al. (41) noted that combining sesame oil and glibenclamide substantially improved the body's antioxidant activity.

## 4 Comparative efficacy of dietary oils and supplementation in managing glycaemic control, lipid profiles, inflammatory, and oxidative markers: insights from recent clinical trials

Nikooyeh et al. (36) examined the effects of ORZ-fortified canola oil, unfortified canola oil, and SO on various cardiometabolic markers. The ORZ-fortified canola oil group experienced significant reductions in fasting blood glucose, HbA1c, and triglycerides compared to the other oils, highlighting its potential for improving glycaemic control in type 2 diabetes. The positive impact on triglycerides also suggests potential benefits for lipid management and cardiovascular risk reduction. This study is notable for its focus on ORZ and its potential benefits beyond traditional oils. The 12-week intervention period, while longer than some studies, may still be insufficient to evaluate long-term benefits or potential side effects. Finally, although a decrease in triglycerides was observed in all groups, this was not significant for unfortified canola oil.

Haldar et al. (39) assessed the effects of a blend of rice bran, flaxseed, and sesame oils compared to refined olive oil on various cardiometabolic markers in borderline hypercholesterolemic individuals. The study reported significant reductions in total cholesterol, LDL-C, triglycerides, and cardiovascular risk ratios and improved blood pressure and blood glucose. However, there was a small but significant increase in body weight, and no significant effects were observed on HDL-C or Apolipoprotein A1 (apoA1) concentrations. The strengths of this study include its parallel design, which allows for a direct comparison between oil blends and a control oil. The reductions in key cardiovascular risk factors and blood pressure underscore the potential benefits of the oil blends. Nonetheless, the study's limitations include a modest increase in body weight, which could affect the overall interpretation of the results. Additionally, the lack of significant changes in HDL-C highlights the need for further research to determine the full impact of these oils on cardiovascular health. A significant limitation of Haldar et al.'s study is that they treated patients with fiber, seed oils, and olive oil. Therefore, it remains unclear whether the observed effects were mainly attributed to the impact of oil or fiber.

Aslam et al. (38) explored the effects of white sesame seed oil compared to soybean oil on glycaemic control and inflammatory and oxidative markers in adults with type 2 diabetes. The study found that sesame seed oil significantly improved glycaemic control, with reductions in blood glucose levels and HbA1c, alongside increases in insulin levels. This suggests that sesame seed oil could benefit diabetes management strategies, potentially offering a natural alternative to conventional therapies. The study also demonstrated that sesame seed oil substantially improved oxidative stress markers. Specifically, Thiobarbituric Acid Reactive Substances (TBARS) levels, a marker of lipid peroxidation, decreased significantly in the sesame oil group, indicating reduced oxidative damage. Additionally, antioxidant enzymes such as Superoxide Dismutase (SOD), Catalase (CAT), and Glutathione Peroxidase (GPx) were elevated in the sesame oil group, reflecting enhanced antioxidant activity. These findings support the potential of sesame seed oil in mitigating oxidative stress, which is a critical factor in the progression of diabetes-related complications. However, there are limitations to consider. The study's open-label design could introduce bias, as participants and researchers were aware of the treatment allocations. This may have influenced the participants' behavior or the researchers' observations.

Khajebishak et al. (42) investigated the impact of PSO supplementation on glycaemic control and gene expression related to glucose metabolism in obese type 2 diabetic patients. The study was a randomized, double-blind clinical trial involving 60 participants randomly assigned to receive either 1 g/day of PSO or placebo capsules for 8 weeks. The results indicated that PSO supplementation led to a significant improvement in fasting blood glucose levels, with a decrease from 161.46 to 143.50 mg/dL and an increase in GLUT-4 gene expression, which is crucial for glucose uptake in cells. The study also observed a slight but non-significant reduction in HbA1c in the PSO group, suggesting potential benefits in long-term glycaemic control. In addition, the PSO group showed a significant improvement in the Quantitative Insulin Sensitivity Check Index (QUICKI), an index of insulin sensitivity, which implies enhanced insulin action. However, there were no significant changes in insulin levels, Homeostatic Model Assessment of Insulin Resistance (HOMA-IR), or HOMA-β between the PSO and placebo groups, indicating that while PSO might improve some aspects of glycaemic control and insulin sensitivity, it does not impact all metabolic parameters uniformly. One of the key strengths of this study is its double-blind design, which minimizes bias and increases the reliability of the results. The significant increase in GLUT-4 gene expression and improvement in QUICKI highlights the potential of PSO as an adjunctive therapy for managing type 2 diabetes, particularly in improving glucose uptake and insulin sensitivity. The study also found no significant changes in insulin levels or HOMA-IR, which suggests that while PSO has some positive effects, its impact on overall insulin resistance and secretion might be limited.

Akrami et al. (45) compared the effects of FO and SO on MetSyn symptoms in individuals with MetSyn. The study found that both oils reduced total cholesterol, LDL-C, and triglycerides. Notably, FO was associated with a significant decrease in Malondialdehyde (MDA), an oxidative stress marker, whereas SO showed no substantial changes in MDA. Both oils effectively improved lipid profiles, but there were no significant differences in fasting blood sugar levels. The findings highlight the potential of both FO and SO in managing lipid levels and reducing oxidative stress. The study's findings underscore the value of dietary oils in managing lipid levels and hypertension. However, the two groups' lack of significant differences in fasting blood sugar levels limits the conclusions about their impact on glycaemic control. Furthermore, the study did not assess the impact on inflammatory and oxidative markers, which could have provided additional insights into the broader health implications of the oils.

Jenkins et al. (37) investigated the impact of a low-GL diet supplemented with canola oil on glycaemic control and lipid profiles in adults with type 2 diabetes. Their study found that the low-GL diet led to significant reductions in HbA1c and improved lipid profiles compared to a whole-grain diet, suggesting that dietary modifications focused on glycaemic load can effectively manage blood glucose levels. Including canola oil in the low-GL diet also positively influences lipid profiles, potentially lowering cardiovascular risk. One of the study's strengths is its clear focus on dietary modification strategies and their impact on multiple health parameters. Additionally, the study did not explore the effects of the diet on inflammatory and oxidative markers, which could provide a more comprehensive view of its health impacts.

Wei et al. (43) conducted a prospective, randomized control trial to examine the combined effects of perilla oil supplementation and exercise on lipid profiles and inflammatory markers in adults with elevated blood lipids. The study involved 36 participants who were divided into three groups: Exercise only (EG), perilla oil only (MG), and a combination of both (EMG). The results showed significant reductions in total cholesterol, triglycerides, and LDL-C in the EMG group compared to the EG and MG groups. Additionally, HDL-C levels increased significantly in both the MG and EMG groups. Notably, the EMG group also experienced significant reductions in inflammatory markers such as high-sensitivity C-Reactive Protein (hs-CRP), Plasminogen Activator Inhibitor-1 (PAI-1), and Tumor Necrosis Factor-alpha (TNF-α) compared to the other groups. The combination of perilla oil and exercise offers enhanced benefits over either intervention alone, particularly in improving lipid profiles and reducing inflammation. The strength of this study lies in its comprehensive approach, combining both dietary and physical activity interventions, which are crucial for managing hyperlipidemia. The study did not assess potential interactions between the exercise regimen and perilla oil supplementation, which could provide further insights into their combined effects.

Alipoor et al. (40) investigated the impact of white sesame seed consumption on lipid profiles and antioxidant markers in hyperlipidemic patients. The study involved 38 randomized participants who received either white sesame seeds (40 g/day) or a control diet for 60 days. The results demonstrated a significant decrease in total cholesterol, LDL-C, and the total cholesterol to HDL-C ratio in the sesame seed group. Additionally, there were improvements in antioxidant markers, with decreased levels of TBARS and increased GPx and SOD activities. The findings suggest that sesame seeds have a beneficial effect on both lipid profiles and oxidative stress. The study's strengths include its clear focus on dietary intervention and its use of both lipid and antioxidant markers. Additionally, the study did not explore potential changes in inflammatory markers, which could offer a more comprehensive view of the health benefits of sesame seeds.

Asghari et al. (44) examined the effects of PSO supplementation on serum triglycerides, HDL-C, and inflammatory markers in adults with elevated cholesterol. The study found that PSO supplementation (800 mg/day for 4 weeks) reduced serum triglycerides and the triglyceride to HDL-C ratio, increasing HDL-C. Despite these positive changes, there were no significant alterations in TNF-α levels, an inflammatory marker. The study's strength lies in its double-blind, placebo-controlled design, which enhances the reliability of the findings. The significant improvements in triglyceride levels and HDL-C suggest that PSO may be effective in managing dyslipidemia. However, the lack of impact on TNF-α levels suggests that while PSO can improve lipid profiles, its effects on inflammation may be limited.

Sankar et al. (41) evaluated the effectiveness of sesame oil alone, glibenclamide alone, and their combination in patients with type 2 diabetes. The combination therapy showed superior improvements in glycaemic control and lipid profiles compared to either treatment alone, indicating that combining therapies can enhance treatment outcomes. The study also observed improvements in antioxidant activity with sesame oil and its combination with glibenclamide, suggesting added benefits in managing oxidative stress. While the study highlights the potential synergistic effects of combining sesame oil with glibenclamide, the open-label design introduces potential bias, as both participants and researchers were aware of the treatment assignments. Despite these limitations, the study provides valuable insights into the potential for combination therapies to improve glycaemic control and antioxidant status, which are crucial for managing diabetes effectively. The focus on oxidative stress adds another layer of understanding to the benefits of sesame oil. However, the study did not address inflammatory markers, which could further elucidate the full spectrum of health benefits.

Mirmiran et al. (46) explored the impact of PSO on lipid profiles and body composition in hyperlipidemic individuals. The study found that PSO supplementation (400 mg twice daily for 4 weeks) reduced triglycerides and improved HDL-C. However, there were no significant changes in serum cholesterol, LDL-C, glucose concentrations, or body composition. The study highlights the potential of PSO to improve triglyceride levels and HDL-C, but its effects on other lipid parameters and metabolic health were not significant. The double-blind design and focus on specific lipid markers add robustness to the findings.

**Figure 2:**
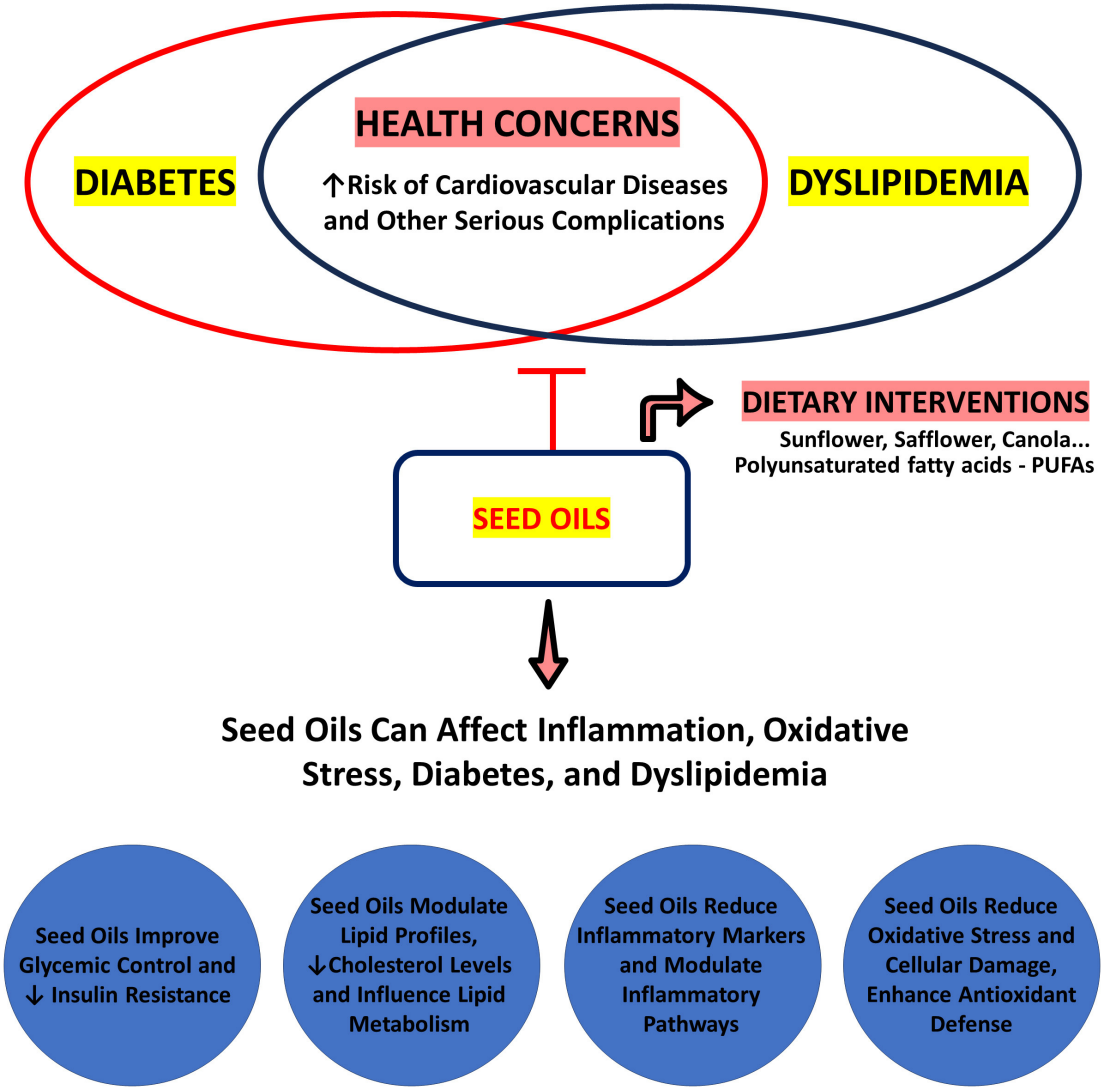
Impact of seed oils on diabetes, dyslipidemia, inflammation, and oxidative stress. Reproduced from Servier Medical Art, licensed under CC BY 4.0.

Figure 2 provides a visual summary of the potential effects of seed oils on key health parameters. The figure illustrates how seed oils may influence diabetes mellitus, dyslipidemia, chronic inflammation, and oxidative stress. Each circle highlights specific actions and outcomes associated with seed oils in these contexts, offering a comprehensive overview of their impact on metabolic and inflammatory pathways. This visual representation aims to clarify the relationships between seed oils and various health markers, setting the stage for a detailed analysis in the subsequent sections.

## 5 Conclusions, limitations, and future research endeavors

This systematic review consolidates evidence from various studies examining the impact of seed oils on glycemic control, lipid profiles, and markers of inflammation and oxidative stress. The findings from these studies highlight the potential therapeutic benefits of different seed oils in managing metabolic disorders, particularly type 2 diabetes and related conditions. PSO has demonstrated potential in improving glycemic control, significantly reducing fasting blood glucose levels, and enhancing GLUT-4 gene expression. However, its impact on other metabolic parameters, such as insulin levels and HOMA-IR, was limited, indicating that while PSO may offer some benefits, it may not uniformly affect all aspects of metabolic health. Sesame seed oil has shown promise in improving glycemic control and reducing oxidative stress, as evidenced by significant decreases in oxidative stress markers and increases in antioxidant enzyme activity. This suggests that sesame seed oil could be valuable to diabetes management strategies. Additionally, studies on flaxseed and sunflower seed oils reveal their positive effects on lipid profiles, with FO particularly notable for reducing oxidative stress markers. The review also underscores the benefits of combining dietary interventions with physical activity, as shown by the study on perilla oil. This integrated approach resulted in improved lipid profiles and reduced inflammatory markers, highlighting the importance of comprehensive lifestyle modifications in managing metabolic conditions.

Nutrigenomics emerges from studying food and dietary components' impacts on gene expression concerning genetic variants and other nutritional factors. It focuses on the interaction between nutrients and functional foods with the genome at the molecular level, allowing insights into the role of specific food compounds or dietary constituents that may influence human health (47). In this scenario, future research into seed oils and their effects on metabolic health should explore genetic studies, as they could reveal how individual genetic variations affect responses to seed oil supplementation since genetic variances may influence the individual response to dietary intakes and supplements (48). By identifying genetic markers associated with varied responses, researchers can develop personalized treatment strategies that optimize the benefits of seed oils based on individual genetic profiles, thereby permitting the practical and objective translation from conventional dietary guidelines to genome-guided nutrition, as evidenced by Lagoumintzis and Patrinos (49). This approach could lead to more targeted and effective interventions, improving outcomes for people with different genetic backgrounds, which is a crucial step in developing alternative and more cost-effective treatment strategies to diabetes and dyslipidemia, as evidenced by recent vital publications (50, 51). Immunological assays represent another essential research direction. These studies can provide detailed insights into how seed oils influence immune system dynamics by assessing changes in immune cell profiles, cytokine levels, and other markers of immune function. As evidenced by Yamasaki et al. (52), dietary interventions with oils derived from pomegranate seeds modulate the immune system and affect the lipid profiles of the treated mice by modulating inflammation derived from immune interactions and adipose tissue dysfunction. Understanding how seed oils modulate inflammation and oxidative stress at the cellular level could uncover new mechanisms of action, potentially identifying novel therapeutic targets and enhancing strategies for managing chronic inflammatory conditions. Diabetes is an inflammatory disease (53), and dyslipidemia strictly correlates with dietary inflammatory indexes (54). Ribonucleic Acid (RNA)-based therapies also offer significant potential for advancing seed oil research. Exploring small interfering RNAs (siRNAs) or messenger RNA (mRNA) therapies to target specific metabolic pathways influenced by seed oils could enhance their therapeutic efficacy since seed oils have already been identified as epigenetic modulators in many diseases (55), including diabetes (56). Such approaches allow for precise modulation of gene expression related to metabolic health, potentially amplifying the beneficial effects of seed oils and mitigating any adverse outcomes. This innovative line of research could lead to more effective treatments that leverage the molecular impacts of seed oils.

Many of the included studies addressed oils against diabetes and dyslipidemia ranging from milligrams to grams treatment options, which limits the understanding of the findings and the generability to the broader population. Because of this, long-term and dose-response studies are essential to fully understanding the sustainability and optimal use of seed oils. Research beyond typical short-term intervention periods is needed to assess seed oil supplementation's long-term benefits and potential risks. Additionally, investigating different dosages will help determine the most effective and safe levels of seed oil intake for various health outcomes. This comprehensive approach will provide valuable insights for clinical practice and public health recommendations. Expanding research to include more diverse and broader populations is also critical. Future studies should involve individuals with various health conditions beyond type 2 diabetes, such as cardiovascular diseases, MetSyn, and obesity. This will improve the generalizability of findings and help identify which groups might benefit most from seed oil interventions. Furthermore, examining the effects of seed oils across different demographic groups—including varying ages, ethnic backgrounds, and lifestyle factors—will ensure that recommendations are relevant and applicable to a broader audience. In summary, pursuing these research directions will deepen our understanding of seed oils' therapeutic potential, enable more personalized treatment strategies, and enhance the applicability of findings across diverse populations and health conditions.

Several limitations were identified in the reviewed studies. Many had relatively short intervention periods, which may not capture the full range of benefits or potential adverse effects of seed oil supplementation. Larger sample sizes were often lacking, which could affect the statistical power and generalizability of the results. Some studies had open-label designs or lacked control for potential biases, which could impact the reliability of the outcomes. Additionally, not all studies assessed a comprehensive range of biomarkers, and many focused on specific populations, limiting the applicability of the findings to the general population. Finally, the included studies varied in dosage and intervention periods, which could impact the comparability and generalizability of the results, making it difficult to draw definitive conclusions. Most included studies did not evaluate the utilized interventions' nutritional composition and chemical profiles. If they had done so, it would have undoubtedly enhanced the strength of the data since most of the included oils might possess similar bioactive compounds and nutritional values. Addressing these limitations through longer, more rigorously designed studies with diverse populations and comprehensive biomarker assessments will be crucial for further elucidating seed oil supplementation's health benefits and potential drawbacks.

## Data Availability

The original contributions presented in the study are included in the article/supplementary material, further inquiries can be directed to the corresponding author.
